# Impact of the right ventricular mechanical pattern assessed by three-dimensional echocardiography on adverse outcomes following cardiac surgery

**DOI:** 10.1038/s41598-025-89122-w

**Published:** 2025-02-15

**Authors:** Marius Keller, Alexandra Fábián, Andrea Bandini, Ádám Szijártó, Zoltán Tősér, Béla Merkely, Tim Heller, Marcia-Marleen Dürr, Peter Rosenberger, Attila Kovács, Harry Magunia

**Affiliations:** 1 Department of Anesthesiology and Intensive Care Medicine, Eberhard Karls University, Hoppe-Seyler-Strasse 3, 72076 Tuebingen, Germany; 2 Heart and Vascular Center, Semmelweis University, 68 Városmajor Street, Budapest, 1122 Hungary; 3 Argus Cognitive, Inc, 35 South Main Street, Hanover, NH 03755 USA

**Keywords:** Transesophageal echocardiography, Three-dimensional echocardiography, Rightventricular function, Risk prediction, Cardiac surgery, Outcomes research, Cardiology, Risk factors

## Abstract

Risk stratification for cardiac surgery is a cornerstone of perioperative management. While the prognostic impact of severe right ventricular (RV) dysfunction is well understood, the added value of the RV mechanical pattern regarding risk prediction remains unknown. We sought to prospectively validate the predictive value of 3D RV mechanics for adverse perioperative outcomes. The clinical and echocardiographic parameters of 439 retrospectively selected patients who underwent various types of cardiac surgery and 3D transesophageal echocardiography were investigated to determine their associations with a composite endpoint of an unfavorable postoperative outcome. Tricuspid regurgitation, 2D RV strains, and 3D measures of left ventricular (LV) and RV function were associated with the composite endpoint. Multivariable logistic regression models revealed that only tricuspid regurgitation, LV ejection fraction and 3D RV global longitudinal strain (GLS) were independently associated with the endpoint. By applying the model to the data of 128 prospectively enrolled patients, only 3D RV GLS remained an independent predictor. A RV GLS cutoff of -17.4% was found to be associated with a 3-fold increased risk for adverse outcomes. This led us to conclude that RV longitudinal deformation derived from 3D echocardiography is predictive of adverse outcomes and should be incorporated in perioperative risk stratification.

**Supplementary Information:**

The online version contains supplementary material available at 10.1038/s41598-025-89122-w.

## Introduction

Granular preoperative risk stratification is of utmost importance for patients undergoing cardiac surgeries. Advances in diagnostic and therapeutic modalities have lowered the incidence of short-term perioperative adverse events over the last decades^1,2^. However, the identification of high-risk individuals is still a clinical issue, as previous stratification tools have become outdated for use with contemporary patient cohorts^3,4^. Conventional transthoracic echocardiography is an integral part of preoperative assessment^5^. It not only provides vast amounts of information, starting from disease diagnosis, for establishing the indication for the intervention, and categorizing the risk based on the severity of the culprit lesion but also quantifies the subsequent damage to the entire cardiopulmonary system. Despite the predictive value of preoperative transthoracic echocardiography, the routine use of intraoperative transesophageal echocardiography (TEE) has severe prognostic implications and is mandatory in current perioperative care^6–8^.

Right ventricular dysfunction is a critical determinant of unfavorable outcomes after cardiac surgeries^9,10^. Although conventional M-mode and two-dimensional (2D) echocardiography-derived morphological and functional metrics have severe shortcomings in characterizing the RV’s complex geometry and contraction patterns, previous studies have suggested their associations with adverse postoperative outcomes^9,11^. Three-dimensional (3D) echocardiography provides significant added diagnostic and prognostic value^12,13^ and is already accessible in the majority of echocardiography labs and operating theatres. Advanced postprocessing tools permit in-depth profiling of RV mechanics by analyzing 3D models of the RV^14^, and novel parameters might enable better risk estimation than conventional metrics. Many available studies demonstrating an association of RV echocardiography with postoperative outcomes lack prospective validation, thus limiting their clinical applicability^11,15,16^.

Our aim was to investigate the association between RV mechanical pattern and short-term postoperative outcomes and validate our findings in a prospective contemporary cohort of cardiac surgery patients using 3D transesophageal echocardiography.

## Methods

### Ethical approval

The study adhered to the principles outlined in the Declaration of Helsinki and received approval from the Ethics Committee of the Medical Faculty at Eberhard Karls University Tuebingen. The data for the retrospective cohort (# IRB 350/2015R) were extracted in an anonymized form, thus exempting the requirement for individual informed consent under German privacy laws. Data for the prospective cohort were collected from patients who provided informed consent and were subsequently enrolled in the institutional 3D echocardiography database (# IRB 613/2019BO2).

### Study design and patient selection

**Fig. 1:**
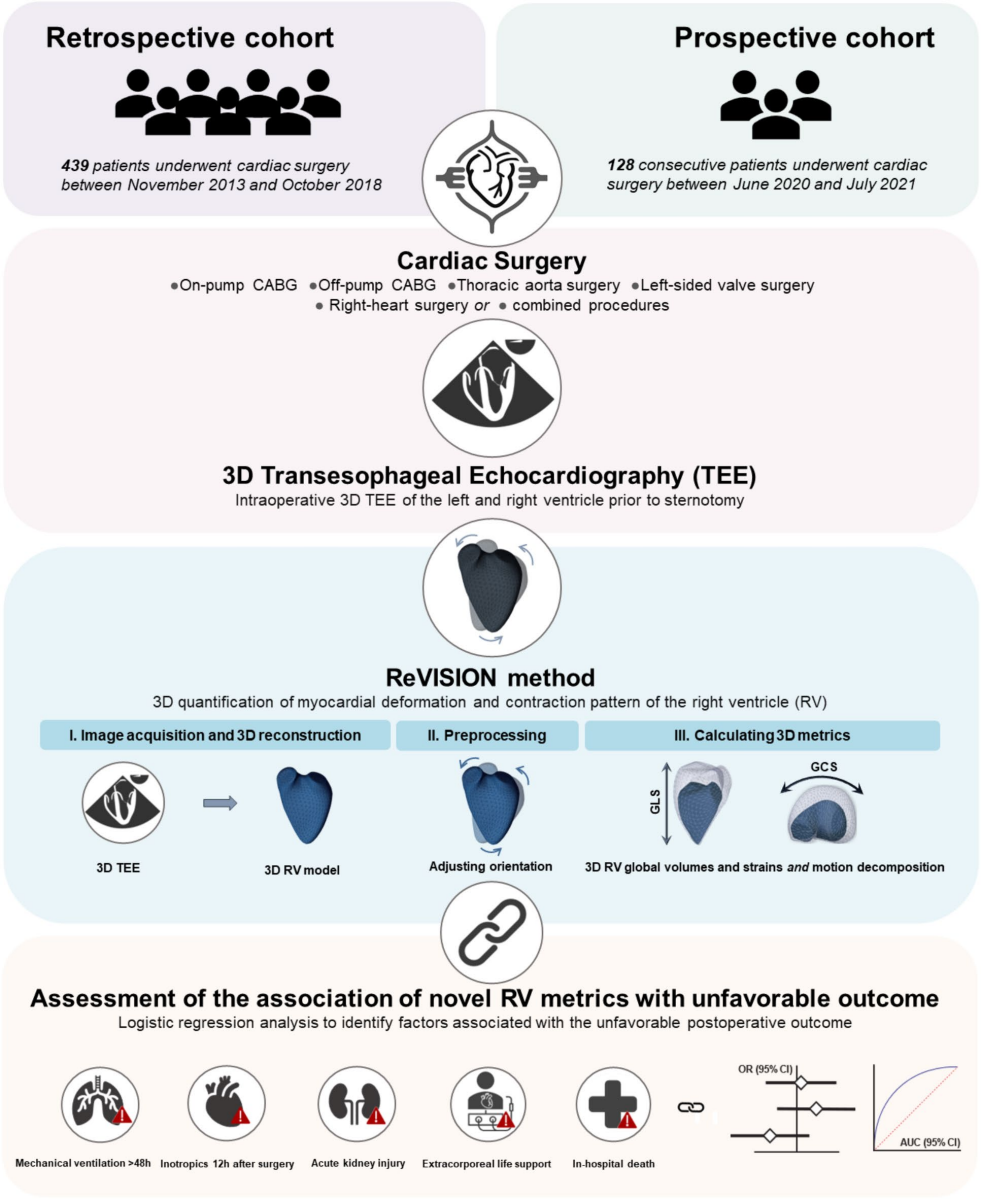
Schematic study overview. A comprehensive display of the study’s design, the investigated cohorts and the methodology. All patients underwent intraoperative transesophageal echocardiography (TEE) after the induction of general anesthesia but prior to sternotomy. Three-dimensional (3D) speckle-tracking-derived characterization of the right ventricle (RV) was performed using the ReVISION method. Together with left ventricular, clinical and surgical data, these parameters were tested for their association with a composite endpoint of adverse postoperative outcomes. Finally, the retrospectively derived observations were validated in prospectively enrolled patients.

This investigation was a single-center study involving two cohorts: one retrospective and the other prospective. The retrospective cohort was used to identify associations between perioperative parameters and outcomes, while the prospective cohort served to internally validate these findings. The study design and methods are outlined in Fig. 1.

Patients who had undergone cardiac surgery between November 2013 and October 2018 were considered for the retrospective cohort if they met specific inclusion criteria: patients older than 18 years at the time of surgery, received intraoperative 3D TEE of the left and right ventricles prior to sternotomy, and underwent TEE according to the institutional standard and had their data stored in an echocardiographic database. The following types of surgeries were considered: left-sided valvular surgery, on-pump coronary artery bypass grafting, off-pump coronary artery bypass grafting, surgery of the thoracic aorta, or combined procedures. Exclusion criteria included incomplete electronic records for clinical data acquisition and preoperative cardiac assist devices (e.g., LVADs or temporary mechanical circulatory support). As recently published, we used a similar patient cohort selected from our retrospective database (*n* = 496) to investigate RV mechanics using a different software approach from the tool used in the present study^16^.

For the prospective cohort, consecutive adult patients undergoing cardiac surgery were enrolled into the institutional echocardiography registry after obtaining ethical approval in June 2020. Informed consent was acquired during preoperative evaluations by anesthesiologists. Only patients who underwent standardized intraoperative TEE, including 3D imaging by specially trained staff were included. Patients who had inappropriate 3D acquisitions, surgeries outside the predefined categories, or preoperative mechanical circulatory support were not included.

### Demographic and clinical data

Demographic and clinical data were extracted from institutional electronic patient records. Parameters included sex, age, weight, height, surgical procedures, duration of cardiopulmonary bypass, estimated glomerular filtration rate (eGFR, MDRD formula), hematocrit, and New York Heart Association (NYHA) functional class. The European System for Cardiac Operative Risk Evaluation II (EuroSCORE II) was calculated, incorporating factors such as pulmonary hypertension, extracardiac arteriopathy, reduced mobility, prior cardiac surgery, chronic lung disease, active endocarditis, critical preoperative state, insulin-dependent diabetes, previous myocardial infarction, the need for hemodialysis, and angina at rest^17^. Surgery was classified as “urgent/emergency” if it needed to be performed within 24 h; otherwise, it was categorized as “elective.” Body mass index (BMI) and body surface area (BSA, calculated using the Du Bois formula) were also determined.

### Three-dimensional echocardiography and established functional parameters

Anesthesia and surgical procedures were conducted in line with the standard protocols of the Department of Anesthesiology & Intensive Care Medicine and the Department of Thoracic & Cardiovascular Surgery at Eberhard Karls University Clinic (Tuebingen, Germany). The institutional protocol stipulates that baseline 3D TEE be performed by trained cardiac anesthesiologists on hemodynamically stable patients after anesthesia induction but before sternotomy. In both the retrospective and prospective cases, the personnel performing 3D TEE were not necessarily involved in anesthesia management, which was carried out by a different anesthetist. Representative 3D images of the right and left ventricles were obtained at frame rates exceeding 20 fps using multi-beat acquisition over four heartbeats. The ultrasound systems and 3D-compatible probes used were commercially available (Philips X7-2t Matrix, Philips Healthcare, Inc., Andover, MA, USA). 3D speckle-tracking analysis of the left ventricle (LV) was performed using proprietary software (4D LV-ANALYSIS, TOMTEC Imaging Systems GmbH, Unterschleissheim, Germany), which provided measurements of end-diastolic volume (LVEDV), end-systolic volume (LVESV), ejection fraction (LVEF), and global longitudinal strain (LV GLS). For right ventricular (RV) assessment, a similar software package (4D RV-Function 3.0, TOMTEC Imaging Systems GmbH, Unterschleissheim, Germany) was used to calculate end-diastolic volume (RVEDV), end-systolic volume (RVESV), and ejection fraction (RVEF). Ventricular volumes were indexed to body surface area. 2D RV free-wall longitudinal strain (FWLS) and global longitudinal strain (GLS) were measured from mid-esophageal four-chamber views using speckle-tracking software (2D CPA, TOMTEC Imaging Systems GmbH, Unterschleissheim, Germany). Image acquisition and measurements adhered to guideline recommendations^18–20^. The severity of tricuspid regurgitation was assessed based on leaflet morphology, the size and area of the color Doppler jet, vena contracta diameter, and right atrial size.

### Detailed characterization of the right ventricular mechanical pattern

**Fig. 2:**
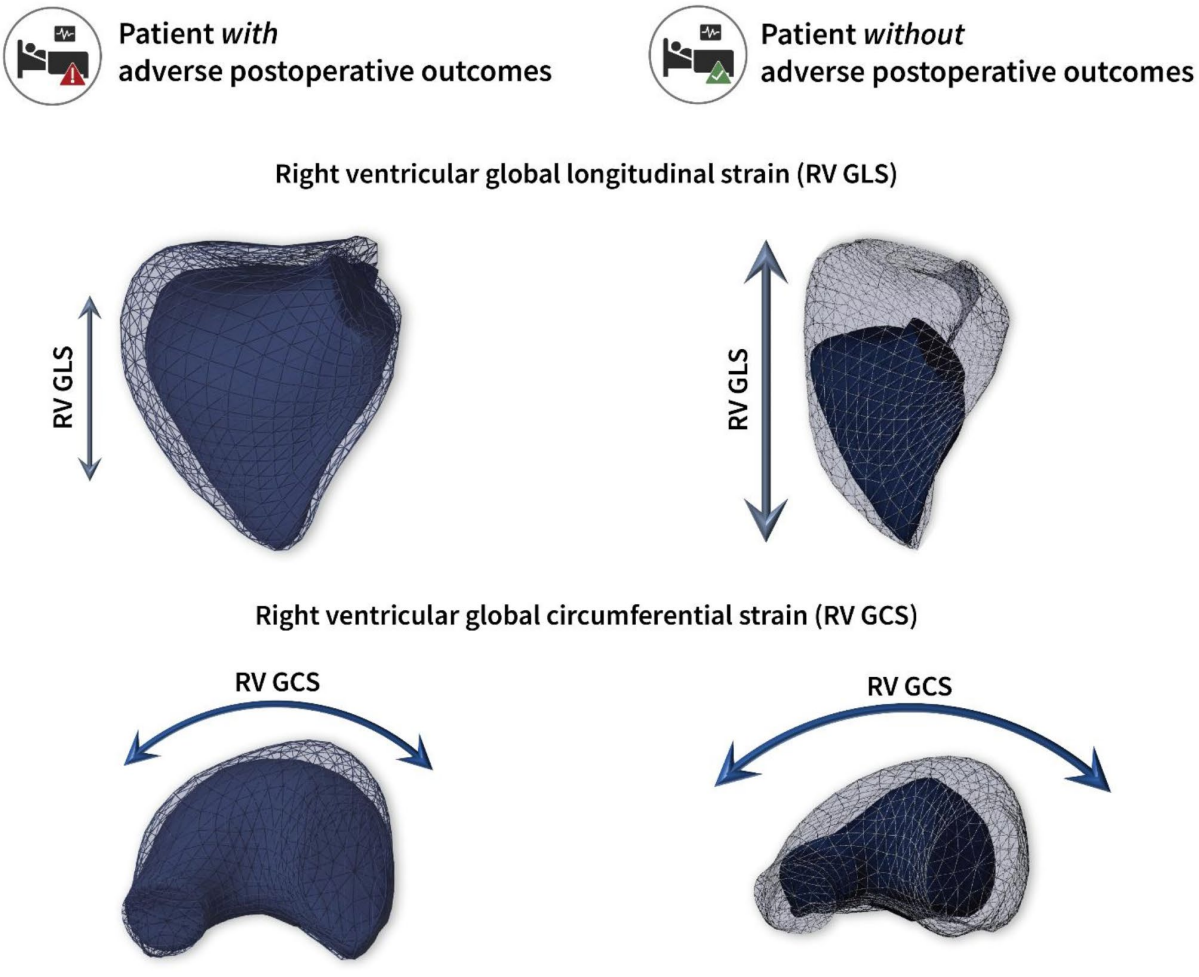
Three-dimensional echocardiographic analysis of the right ventricular mechanical pattern. Mesh models of the right ventricle (RV) derived from transesophageal three-dimensional speckle-tracking echocardiography underwent postprocessing using the ReVISION method. A representative patient from the prospective cohort experiencing adverse postoperative outcomes (left) shows severely reduced RV function compared to a patient from the same cohort with an uneventful postoperative course (right). *(Top row: anterior views of the RV*,* bottom row: basal views of the RV)*

The ReVISION software (Argus Cognitive, Inc., Lebanon, NH, USA) was employed to quantify the three primary functional components of RV performance. A 3D mesh model, exported from the 4D RV-Function software, was reoriented using an automated method to identify the longitudinal (from the tricuspid annulus to the apex), radial (perpendicular to the interventricular septum), and anteroposterior (parallel to the interventricular septum) axes. Motion decomposition was then conducted along these axes using a vertex-based approach to calculate the contributions of each motion component, including longitudinal ejection fraction (LEF), radial ejection fraction (REF), and anteroposterior ejection fraction (AEF), as previously described^21^. Additionally, the software computed 3D RV global longitudinal strain (GLS), global circumferential strain (GCS), and global area strain (GAS) (Fig. 2).

### Outcome definition

The primary endpoint was defined as a composite of adverse postoperative outcomes (APOs), which included in-hospital mortality, the need for veno-arterial extracorporeal life support, prolonged mechanical ventilation exceeding 48 h, the requirement for inotropics 12 h after surgery, and/or acute kidney injury (AKI), characterized by a serum creatinine increase of > 0.3 mg/dl within the first two days of ICU admission or the initiation of renal replacement therapy during the ICU stay.

### Statistical analysis

The statistical analyses were conducted using SPSS (v22, IBM, Armonk, NY, USA) and R (version 3.6.2, R Foundation for Statistical Computing, Vienna, Austria). Continuous variables were expressed as mean ± standard deviation (SD), or median (interquartile range). Categorical variables were reported as frequencies and percentages. The normality of continuous variables was assessed using the Shapiro‒Wilk test. Outcome group comparisons were performed using unpaired Student’s t-tests for normally distributed continuous variables, Mann‒Whitney U tests for non-normally distributed continuous variables, and Chi-squared or Fisher’s exact tests for categorical variables, as appropriate. Logistic regression analysis was used to identify risk factors associated with the composite outcome, with odds ratios (ORs) and their 95% confidence intervals (CIs) reported. In the retrospective cohort, variables of clinical interest from univariable logistic regression with *p* < 0.1 were compared using the Akaike information criterion (AIC) to identify the best-fitting model, and those with lower AIC values were included in multivariable logistic regression models. A similar model was constructed using the prospective cohort for internal validation. Collinearity was evaluated using the variance inflation factor (VIF), considering values > 3 as indicative of excessive collinearity. Receiver operating characteristic (ROC) curves were generated to assess the discriminative power of 3D echocardiographic parameters for predicting the composite endpoint of APOs. A two-sided p-value of < 0.05 was considered statistically significant.

## Results

### Retrospective cohort: patient characteristics, echocardiographic assessment and outcomes

Retrospective patient selection resulted in the inclusion of 439 patients undergoing surgery between June 2015 and October 2018 (Supplementary Figure S1). A total of 208 (47%) of these patients reached the endpoint (Supplementary Table S1). Baseline demographics, clinical characteristics and parameters of LV function stratified by the experience of APOs are displayed in Table 1 (left columns). Tricuspid regurgitation was more likely to be present in the group who reached the endpoint. Patients with APOs underwent significantly longer durations of cardiopulmonary bypass. Patients undergoing off-pump coronary bypass surgery were less likely to experience APOs.

**Table 1:** Baseline clinical characteristics of the study cohorts depending on the occurrence of adverse postoperative outcomes (APOs).

	Retrospective cohort (*n* = 439)		*p*-value	Prospective cohort (*n* = 128)		*p*-value
	*no* APOs (*n* = 231)	APOs (*n* = 208)		*no* APOs (*n* = 100)	APOs (*n* = 28)	
*Clinical characteristics*						
Age, years	65.0 ± 13.9	65.1 ± 13.6	0.908	64.0 ± 11.7	67.6 ± 11.0	0.155
Female, n	73 (31.6)	51 (24.5)	0.099	18 (18.0)	6 (21.4)	0.681
Body mass index, kg/m²	27.0 ± 4.7	27.7 ± 5.0	0.143	28.3 ± 6.9	28.8 ± 6.1	0.715
eGFR, ml/min	88.1 ± 31.9	82.6 ± 38.7	0.107	82.4 ± 24.7	72.1 ± 26.9	0.058
Hematocrit, %	38.0 ± 4.9	36.9 ± 6.3	0.058	39.5 ± 4.3	37.7 ± 5.1	0.068
Pulmonary hypertension*, n	27 (11.7)	38 (18.3)	0.053	3 (3.0)	4 (14.3)	0.020
Significant tricuspid regurgitation (grade ≥ 2), n	19 (8.2)	37 (17.8)	0.003	7 (7.0)	5 (17.9)	0.081
EuroSCORE II, %	3.17 (1.54–5.76)	3.43 (1.58–8.10)	0.184	1.55 (0.94–3.26)	3.56 (2.19–5.29)	0.001
NYHA functional class, n						
I	51 (22.1)	42 (20.2)	0.629	46 (46.0)	8 (28.6)	0.099
II	78 (33.8)	76 (36.5)	0.543	32 (32.0)	5 (17.9)	0.145
III	70.0 (30.3)	61 (29.3)	0.823	20 (20.0)	13 (46.4)	0.005
IV	32 (13.9)	29 (13.9)	0.978	1 (1.0)	2 (7.1)	0.056
*Cardiac surgery*						
Type of surgery						
On-pump coronary artery bypass grafting, n	31 (13.4)	33 (15.9)	0.468	11 (11)	5 (17.9)	0.332
Off-pump coronary artery bypass grafting, n	65 (28.1)	27 (13.0)	< 0.001	33 (33.0)	4 (14.3)	0.054
Left-sided valve surgery, n	65 (28.1)	57 (27.4)	0.864	28 (28.0)	7 (25.0)	0.753
Thoracic aortic surgery, n	8 (3.5)	8 (3.8)	0.831	0 (0.0)	1 (3.6)	0.058
Combined procedures, n	57 (24.7)	78 (37.5)	0.004	27 (27.0)	11 (39.3)	0.209
Right-heart surgery, n	5 (2.2)	5 (2.4)	0.867	1 (1.0)	0 (0.0)	0.595
Priority						
Elective, n	176 (76.2)	157 (75.5)	0.862	87 (87.0)	22 (78.6)	0.268
Urgent/emergency, n	55 (23.8)	51 (24.5)	0.862	13 (13.0)	6 (21.4)	0.268
Cardiopulmonary bypass time, min	116 (93–140)	142 (113–174)	< 0.001	106 (80–130)	116 (102–149)	0.067
*Intraoperative 3D LV speckle-tracking echocardiography*						
LVEDV, ml	153.1 ± 59.1	174.8 ± 71.3	0.001	122.6 ± 44.5	139.4 ± 43.2	0.079
LVEDVi, ml/m²	80.6 ± 31.7	88.8 ± 33.8	0.010	62.7 ± 21.9	69.2 ± 20.6	0.162
LVESV, ml	91.1 ± 45.3	111.6 ± 63.0	< 0.001	65.2 ± 36.2	86.4 ± 47.3	0.012
LVESVi, ml/m²	48.0 ± 24.3	56.6 ± 30.2	0.001	33.2 ± 17.3	42.5 ± 22.9	0.021
LVEF, %	41.9 ± 11.9	38.2 ± 13.7	0.003	46.8 ± 14.4	40.8 ± 18.2	0.069
LV GLS, %	-13.6 ± 5.1	-12.5 ± 5.8	0.039	-13.9 ± 6.3	-12.7 ± 7.0	0.366
*Intraoperative 2D RV speckle-tracking echocardiography*						
2D RV FWLS, %	-19.5 ± 8.2	-17.5 ± 7.8	0.009	-26.3 ± 6.5	-23.7 ± 10.8	0.113
2D RV GLS, %	-16.3 ± 5.8	-14.2 ± 5.5	< 0.001	-20.7 ± 5.4	-18.6 ± 9.5	0.132

Values are means ± standard deviations, medians (interquartile ranges) or n (%).

2D = two-dimensional, 3D = three-dimensional, APOs = adverse postoperative outcomes, eGFR = estimated glomerular filtration rate, EuroSCORE = European System for Cardiac Operative Risk Evaluation, LV GLS = left ventricular global longitudinal strain, LVEDV(i) = left ventricular end-diastolic volume (index), LVESV(i) = left ventricular end-systolic volume (index), LVEF = left ventricular ejection fraction, NYHA = New York Heart Association, *defined as systolic pulmonary artery pressure > 30mmHg, RV FWLS = right ventricular free wall longitudinal strain, RV GLS = right ventricular global longitudinal strain.

Intraoperative 3D speckle-tracking echocardiography revealed impaired LV function (reflected by increased LV volumes and reduced LVEF and LV GLS) and impaired RV function (reflected by increased RV end-systolic volumes and reduced RVEF, Table 2, left columns) in patients who experienced APOs. Novel RV characterization using ReVISION showed marked reductions in RV strains (RV GLS, RV GCS and RV GAS) and motion decomposition metrics (LEF and AEF) of patients experiencing APOs (Table 2, left columns).

**Table 2:** Novel 3D right ventricular parameters and volumes depending on the occurrence of adverse postoperative outcomes (APOs).

	Retrospective cohort (*n* = 439)		*p*-value	Prospective cohort (*n* = 128)		*p*-value
	*no* APOs (*n* = 231)	APOs (*n* = 208)		*no* APOs (*n* = 100)	APOs (*n* = 28)	
Global metrics						
RVEDV, ml	146.2 ± 46.5	152.9 ± 51.7	0.153	130.7 ± 31.4	152.3 ± 47.3	0.005
RVEDVi, ml/m²	75.7 ± 22.5	77.6 ± 24.8	0.403	66.8 ± 16.6	75.2 ± 19.5	0.024
RVESV, ml	88.9 ± 35.4	98.3 ± 39.1	0.009	72.8 ± 24.0	96.9 ± 42.2	< 0.001
RVESVi, ml/m²	46.0 ± 17.7	49.8 ± 18.9	0.032	37.0 ± 11.7	47.3 ± 17.1	< 0.001
RVEF, %	39.8 ± 9.3	36.4 ± 9.3	< 0.001	44.9 ± 8.0	37.9 ± 10.4	< 0.001
RV GLS, %	-14.8 ± 4.7	-12.7 ± 4.6	< 0.001	-17.3 ± 4.1	-14.3 ± 4.8	0.001
RV GCS, %	-14.4 ± 5.4	-13.3 ± 5.1	0.032	-17.5 ± 4.7	-14.3 ± 4.9	0.002
RV GAS, %	-26.5 ± 7.5	-23.8 ± 7.0	< 0.001	-30.0 ± 6.3	-25.1 ± 7.8	0.001
Motion decomposition metrics						
LEF, %	16.3 ± 5.5	14.1 ± 5.5	< 0.001	17.5 ± 5.2	14.9 ± 5.8	0.024
LEF/RVEF	0.41 ± 0.11	0.39 ± 0.13	0.056	0.39 ± 0.09	0.39 ± 0.10	0.941
AEF, %	17.3 ± 6.1	15.4 ± 5.7	0.001	22.1 ± 6.3	17.7 ± 7.3	0.002
AEF/RVEF	0.43 ± 0.11	0.42 ± 0.11	0.322	0.49 ± 0.10	0.46 ± 0.13	0.121
REF, %	15.9 ± 8.0	14.6 ± 8.1	0.116	19.5 ± 7.5	15.5 ± 7.1	0.012
REF/RVEF	0.39 ± 0.16	0.39 ± 0.17	0.952	0.43 ± 0.14	0.40 ± 0.13	0.360

Values are means ± standard deviations.

AEF = anteroposterior ejection fraction, APOs = adverse postoperative outcomes, LEF = longitudinal ejection fraction, REF = radial ejection fraction, RVEDV(I) = right ventricular end-diastolic volume (index), RVEF = right ventricular ejection fraction, RVESV(I) = right ventricular end-systolic volume index, RV GAS = right ventricular global area strain, RV GCS = right ventricular global circumferential strain, RV GLS = right ventricular global longitudinal strain.

Among the investigated clinical parameters, significant tricuspid regurgitation was associated with the occurrence of APOs in univariable logistic regression analysis (Table 3). Furthermore, all investigated 3D LV and the majority of the 3D RV echocardiographic parameters showed a significant association with APOs, including RVESVi, RVEF, RV GLS, RV GCS, RV GAS, LEF, and AEF, as well as 2D RV FWLS and 2D GLS. Multivariable logistic regression analysis was performed using variables of clinical interest from the univariable analysis with *p* < 0.1 and lower absolute AIC values (Supplementary Table S2). All parameters that were included in the multivariable model, namely, the presence of significant tricuspid regurgitation, LVEF and RV GLS, were found to be independently associated with the endpoint (Table 4).

**Table 3:** Univariable logistic regression for the composite endpoint of an unfavorable postoperative outcome in the retrospective cohort.

Variable	Composite endpoint	
	OR [95% CI]	p-value
Clinical characteristics		
Age, years	1.001 [0.987–1.015]	0.908
eGFR, ml/min	0.996 [0.990–1.001]	0.109
Hematocrit, %	0.968 [0.936–1.001]	0.059
NYHA > II	0.965 [0.661–1.407]	0.852
Pulmonary hypertension*	1.689 [0.990–2.880]	0.054
Significant tricuspid regurgitation (grade ≥ 2)	2.414 [1.340–4.350]	0.003
Urgent/emergency procedure	1.039 [0.671–1.610]	0.862
3D LV echocardiographic parameters		
LVEDVi, ml/m²	1.008 [1.002–1.014]	0.011
LVESVi, ml/m²	1.012 [1.004–1.019]	0.002
LVEF, %	0.978 [0.963–0.992]	0.003
LV GLS, %	1.037 [1.002–1.074]	0.040
2D RV speckle-tracking echocardiography		
2D RV FWLS, %	1.032 [1.008–1.057]	0.010
2D RV GLS, %	1.067 [1.032–1.104]	< 0.001
ReVISION-derived 3D RV parameters		
RVEDVi, ml/m²	1.003 [0.995–1.012]	0.402
RVESVi, ml/m²	1.012 [1.001–1.022]	0.034
RVEF, %	0.962 [0.942–0.982]	< 0.001
RV GLS, %	1.105 [1.059–1.153]	< 0.001
RV GCS, %	1.040 [1.003–1.078]	0.033
RV GAS, %	1.054 [1.026–1.083]	< 0.001
LEF, %	0.928 [0.896–0.962]	< 0.001
LEF/RVEF	0.205 [0.040–1.052]	0.058
AEF, %	0.948 [0.918–0.980]	0.001
AEF/RVEF	0.424 [0.078–2.308]	0.321
REF, %	0.981 [0.959–1.005]	0.117
REF/RVEF	1.036 [0.331–3.237]	0.952

2D = two-dimensional, 3D = three-dimensional, AEF = anteroposterior ejection fraction, CI = confidence interval, eGFR = estimated glomerular filtration rate, LEF = longitudinal ejection fraction, LV GLS = left ventricular global longitudinal strain, LVEDV(i) = left ventricular end-diastolic volume (index), LVESV(i) = left ventricular end-systolic volume (index), LVEF = left ventricular ejection fraction, NYHA = New York Heart Association, OR = odds ratio, REF = radial ejection fraction, RVEDV(i) = right ventricular end-diastolic volume (index), RVEF = right ventricular ejection fraction, RVESV(i) = right ventricular end-systolic volume index, RV FWLS = right ventricular free wall longitudinal strain, RV GAS = right ventricular global area strain, RV GCS = right ventricular global circumferential strain, RV GLS = right ventricular global longitudinal strain, *defined as systolic pulmonary artery pressure > 30mmHg.

**Table 4:** Multivariable logistic regression for the composite endpoint of an unfavorable postoperative outcome in the retrospective cohort.

Variable	Composite endpoint	
	OR [95% CI]	p-value
Significant tricuspid regurgitation (grade ≥ 2)	2.497 [1.362–4.578]	0.003
LVEF	0.988 [0.972–1.005]	0.005
(3D) RV GLS	1.094 [1.045–1.145]	< 0.001

3D = three-dimensional, CI = confidence interval, LVEF = left ventricular ejection fraction, OR = odds ratio, RV GLS = right ventricular global longitudinal strain.

**Fig. 3:**
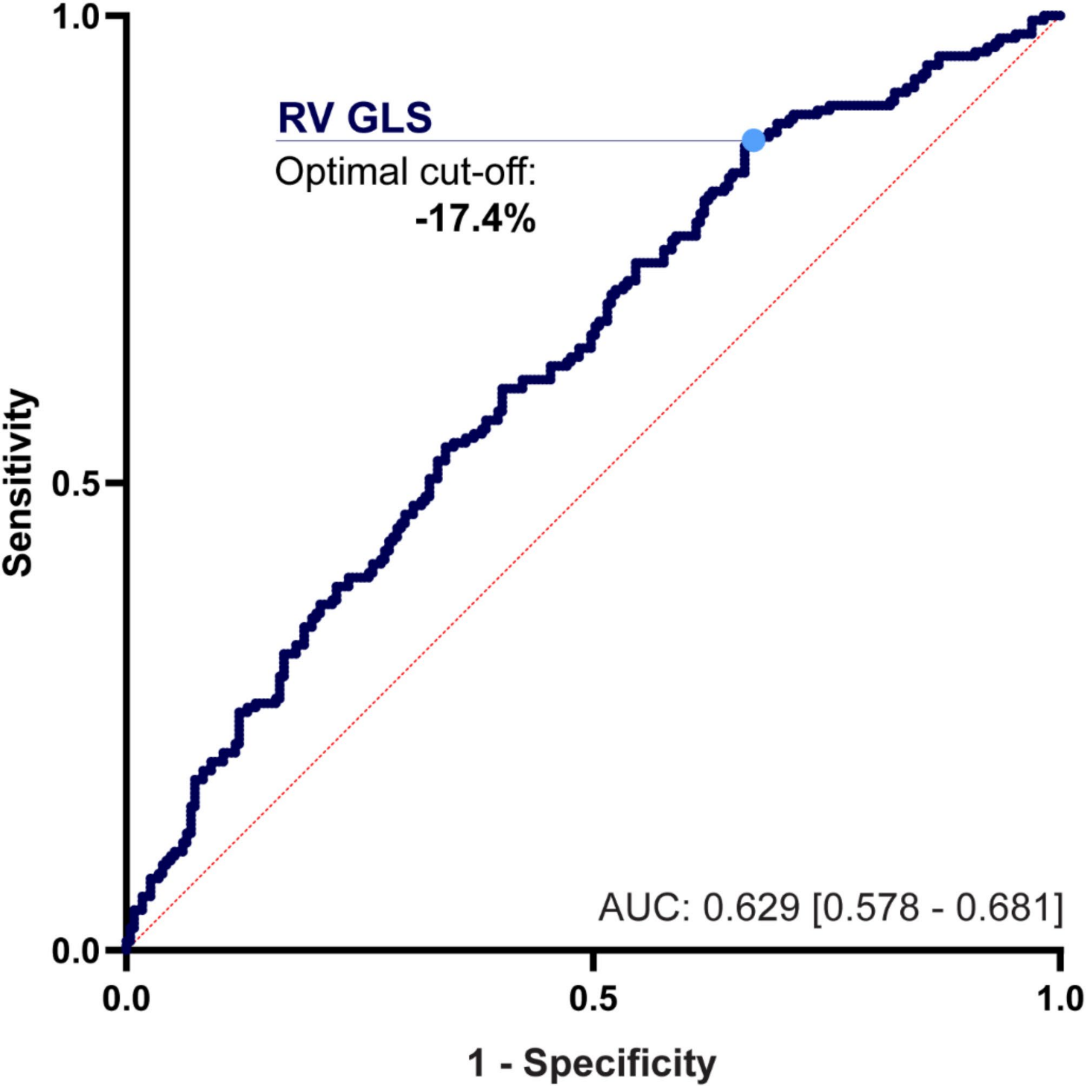
Receiver operating characteristic curve of right ventricular global longitudinal strain (RV GLS). Receiver operating characteristic analysis of RV GLS was performed in the retrospective patient cohort using the composite endpoint of adverse postoperative events. The optimal cutoff value was calculated at -17.4%. *AUC = area under the curve*.

Receiver operating characteristic analysis revealed an optimal RV GLS cutoff of -17.4% for the discrimination of patients with or without APOs, with an area under the curve of 0.629 (Fig. 3). The area under the curve of RV GLS was higher than that of LVEF, LV GLS and RVEF (Supplementary Table S3). According to univariable logistic regression, an RV GLS worse than − 17.4% was associated with in an approximately 3-fold increase in the risk for APOs (OR 3.352 [95% CI 2.057–5.461], *p* < 0.001).

### Prospective cohort: endpoint incidence and validation

Prospective patient enrollment and data collection resulted in the final inclusion of 128 patients between June 2020 and July 2021 (Supplementary Figure S2), and 28 patients (22%) experienced APOs (Supplementary Table S4). The demographic, clinical and LV characteristics of the prospective cohort are listed in Table 1 (right columns). Pulmonary hypertension and NYHA functional class III were more likely to be present in the unfavorable outcome group, whereas the presence of significant tricuspid regurgitation did not differ significantly. The time on cardiopulmonary bypass was not significantly longer in the unfavorable outcome group.

Strikingly, systolic volumes were the only LV parameters significantly different between the outcome groups, showing increased values in patients experiencing APOs. LVEF was reduced in these patients but was not significantly different (*p* = 0.069) between the outcome groups. However, 3D mesh-derived RV metrics (Table 2, right columns) reflecting volumes (RVEDVi and RVESVi) and systolic function (RVEF, RV GLS, RV GCS, RV GAS) differed significantly between the outcome groups. Similar to the retrospective cohort, motion decomposition revealed significant reductions in all three RV motion components (LEF, AEF and REF) in patients who experienced the APOs.

In the univariable logistic regression analysis (Table 5), a NYHA functional class > II and the presence of pulmonary hypertension were significantly associated with the endpoint, as well as LVESVi and the majority of the novel 3D RV metrics (RVEDVi, RVESVi, RVEF, RV GLS, RV GCS, RV GAS, LEF, AEF, REF). In summary, the results of the prospective cohort were comparable to those of the retrospective cohort.

**Table 5:** Univariable logistic regression for the composite endpoint of an unfavorable postoperative outcome in the prospective cohort.

Variable	Composite endpoint	
	OR [95% CI]	p-value
Clinical characteristics		
Age, years	1.029 [0.989–1.071]	0.156
eGFR, ml/min	0.983 [0.966–1.001]	0.061
Hematocrit, %	0.920 [0.841–1.007]	0.071
NYHA > II	4.286 [1.768–10.387]	0.001
Pulmonary hypertension*	5.389 [1.130-25.702]	0.035
Significant tricuspid regurgitation (grade ≥ 2)	2.888 [0.840–9.931]	0.092
Urgent/emergency procedure	1.825 [0.623–5.345]	0.272
3D LV echocardiographic parameters		
LVEDVi, ml/m²	1.013 [0.995–1.032]	0.166
LVESVi, ml/m²	1.023 [1.002–1.045]	0.030
LVEF, %	0.975 [0.948–1.002]	0.069
LV GLS, %	1.031 [0.965–1.102]	0.364
2D RV speckle-tracking echocardiography		
2D RV FWLS, %	1.049 [0.989–1.113]	0.114
2D RV GLS, %	1.058 [0.983–1.138]	0.132
ReVISION-derived 3D RV parameters		
RVEDVi, ml/m²	1.026 [1.003–1.051]	0.029
RVESVi, ml/m²	1.053 [1.021–1.087]	0.001
RVEF, %	0.917 [0.871–0.964]	< 0.001
RV GLS, %	1.176 [1.060–1.305]	0.002
RV GCS, %	1.157 [1.050–1.276]	0.003
RV GAS, %	1.115 [1.042–1.193]	0.002
LEF, %	0.913 [0.843–0.990]	0.027
LEF/RVEF	1.186 [0.013-107.128]	0.941
AEF, %	0.896 [0.834–0.963]	0.003
AEF/RVEF	0.039 [0.001–2.423]	0.124
REF, %	0.928 [0.874–0.986]	0.015
REF/RVEF	0.241 [0.011–5.067]	0.360

2D = two-dimensional, 3D = three-dimensional, AEF = anteroposterior ejection fraction, CI = confidence interval, eGFR = estimated glomerular filtration rate, LEF = longitudinal ejection fraction, LV GLS = left ventricular global longitudinal strain, LVEDV(i) = left ventricular end-diastolic volume (index), LVESV(i) = left ventricular end-systolic volume (index), LVEF = left ventricular ejection fraction, NYHA = New York Heart Association, OR = odds ratio, REF = radial ejection fraction, RVEDV(i) = right ventricular end-diastolic volume (index), RVEF = right ventricular ejection fraction, RVESV(i) = right ventricular end-systolic volume index, RV FWLS = right ventricular free wall longitudinal strain, RV GAS = right ventricular global area strain, RV GCS = right ventricular global circumferential strain, RV GLS = right ventricular global longitudinal strain, *defined as systolic pulmonary artery pressure > 30mmHg.

To perform internal validation, a similar multivariable logistic regression model was constructed as described above, using variables of clinical interest from the univariable analysis with *p* < 0.1 and lower absolute AIC values (Supplementary Table S5). NYHA functional class > II showed a better model fit than the presence of significant tricuspid regurgitation based on AIC values, whereas LVESVi and RVEF also showed a better model fit. However, including the parameters previously identified in the multivariable model of the retrospective cohort (tricuspid regurgitation, LVEF and RV GLS), only RV GLS remained independently associated with the endpoint in the prospective cohort (Table 6 and Supplementary Table S6). Of note, another multivariable model was constructed containing NYHA functional class > II, LVEF and RV GLS, in which only NYHA > II and RV GLS showed significant associations with APOs (Supplementary Table S7). Application of the previously derived RV GLS cutoff of -17.4% was found to be associated with a 3-fold increased risk for APOs in the prospective cohort (OR 3.000 [95% CI 1.171–7.687], *p* = 0.022).

**Table 6:** Multivariable logistic regression for the composite endpoint of an unfavorable postoperative outcome in the prospective cohort.

Variable	Composite endpoint	
	OR [95% CI]	p-value
Significant tricuspid regurgitation (grade ≥ 2)	2.112 [0.560–7.955]	0.269
LVEF	0.990 [0.961–1.020]	0.504
(3D) RV GLS	1.151 [1.032–1.284)	0.011

3D = three-dimensional, CI = confidence interval, LVEF = left ventricular ejection fraction, OR = odds ratio, RV GLS = right ventricular global longitudinal strain.

## Discussion

In our current study, we analyzed a large retrospective cohort of patients undergoing cardiac surgery to explore the associations between advanced echocardiographic parameters and adverse clinical outcomes. To the best of our knowledge, to date, this is the largest cohort of patients undergoing cardiac surgery investigated by 3D transesophageal echocardiography. We also aimed to validate the findings derived from a retrospective cohort in a prospective cohort of patients. According to our results, advanced 3D analysis of the RV provides powerful metrics that are associated with perioperative adverse outcomes, and this predictive value is independent of LV function and other clinical characteristics. Among the different metrics, the parameters of RV longitudinal shortening exhibited the strongest correlation with adverse outcomes, especially 3D RV GLS, which demonstrated superior predictive value compared to 2D RV longitudinal strains. Future investigations are necessary to externally validate these findings and confirm their generalizability to patients undergoing cardiac surgery. Doing so, a major challenge of such studies is the choice of appropriate endpoints. Similarly to previous reports^22^, we chose a composite of short-term adverse postoperative events. Other studies typically use different types of mortality to define in-hospital^2^ or 30-day outcome^11,23^. A recent consensus document regarding standardized endpoints in perioperative medicine pointed out the definition of “major adverse cardiovascular event (MACE)” used in nearly all major clinical trials lacks uniformity, hindering inter-study comparability^24^. The choice of a composite endpoint usually comprises clinically relevant adverse events beyond mortality (e.g., the need for mechanical circulatory support), impeding the need for significantly higher patient numbers due to procedures with low perioperative mortalities.

Perioperative RV dysfunction increases the risk of mortality. It occurs in a substantial number of planned cardiac surgical cases^25,26^. Due to the complexity of the procedures and preexisting cardiac or pulmonary conditions, the reasons for RV dysfunction are manifold. They can be associated with chronic RV pathologies and/or the procedure itself, such as RV dysfunction due to incomplete myocardial protection with cardioplegia. While some forms of RV dysfunction are transient – as in temporary RV failure due to coronary air embolism – others can be permanent. Irrespective of the underlying cause, perioperative RV dysfunction has an undisputed impact on perioperative patient prognosis following cardiac surgery^9,11,27^. However, while perioperative RV dysfunction appears common, a clear definition based on a single parameter remains unknown^28^. Findings characterizing RV longitudinal function and associating its impairment with adverse postoperative outcomes are not novel^16^. The more sophisticated the assessment of RV function is – e.g., quantifying RV deformation or employing 3D studies – the more reliable the assumptions with regard to patient risk^15^. If such parameters are applied using (yet to be established) cutoff values – e.g., RV GLS of approximately − 17% according to our results – the identification of patients at a high risk for unfavorable outcomes following cardiac surgery may be facilitated. Moreover, this could influence clinical decision-making: high-risk patients potentially benefit from off-pump coronary bypass techniques or the avoidance of antegrade cardioplegia (as retrograde cardioplegia leads to inadequate protection of the RV^29^), or require advanced intraoperative hemodynamic management (e.g., right heart catheterization) or postoperative care (e.g., prolonged ICU therapy or admission to specialized centers). The potential role of RV GLS-based guidance on perioperative interventions needs to be investigated in appropriate trials. Regarding risk assessment, currently established risk scores, such as the EuroSCORE II or the Society of Thoracic Surgeons Risk Score, work fairly well on the general populations they were derived from^17,30^. While our findings stress the role of baseline RV function on patient outcome, they add to the unveiling of the scores’ blind spot, as none of them contain direct measures of baseline RV function to date.

Transesophageal echocardiography has become an essential component of standard perioperative care, as its benefits extend beyond postoperative diagnosis of RV dysfunction to include the evaluation of preoperative risks for unfavorable outcomes^31^. Ejection fraction is the primary echocardiographic measure of ventricular function. However, due to the nature of its calculation, which heavily relies on geometry and its subsequent dependence on loading conditions, several limitations need to be addressed^32^. Therefore, advanced imaging methods pursue novel metrics that overcome these shortcomings by being more reflective of intrinsic cardiac contractility or by identifying subclinical dysfunction through the assessment of changes in contraction patterns. Global longitudinal strain by 2D speckle tracking echocardiography has an established added prognostic value compared to LVEF^33^. This result mainly originates from the fact that longitudinal deformation is more sensitive to subtle changes in contractility than a global volumetric response. Despite the fact that 3D echocardiography-derived RVEF has a superior prognostic value compared to conventional 2D RV functional parameters (i.e., tricuspid annular plane systolic excursion [TAPSE], fractional area change, free wall longitudinal strain)^12^, we can assume that an in depth, 3D-based characterization of the more complex RV contraction patterns can unveil a similar added value. Tokodi et al. found that preoperatively increased longitudinal shortening (LEF) is predictive of perioperative RV dysfunction in primary mitral regurgitation patients with a maintained baseline LV and RVEF undergoing mitral valve replacement or repair^34^. Surkova and colleagues found in a mixed left-heart disease population that specific changes in RV contraction patterns are associated with future adverse events even in those cases where RVEF is normal^35^. Our results confirm the aforementioned findings in our cohort of patients undergoing cardiac surgery: measures of longitudinal shortening exhibited the strongest association with adverse perioperative outcomes, systematically overcoming global functional measures of both the LV and the RV.

Despite different loading conditions, specific disease states can induce various changes in the RV contraction pattern and sensitivity, and thus, the superior value of longitudinal RV shortening can be justified. First, the subendocardial layer of the RV myocardium is dominantly longitudinally oriented, and this layer is affected first by wall stress induced by overload conditions^36^. Second, longitudinal RV shortening is coupled with both LV longitudinal shortening and atrial mechanics, meaning that LV dysfunction and elevated RA pressures will impact this motion direction first^37^. Although longitudinal RV shortening can be measured by more simplistic measures, such as TAPSE or 2D free-wall longitudinal strain, these are derived from a single two-dimensional plane (four-chamber view). However, the RV free wall has a huge surface and a complex geometry that can be comprehensively investigated only by 3D echocardiography. Concerning LV GLS calculation, three different tomographic planes are used, resulting in a powerful global metric of LV systolic function^33^. However, 2D speckle tracking-derived RV (or just free wall) longitudinal strain is derived from a single plane with an inherent risk for significant loss of information^38^. Interestingly, while longitudinal shortening was significantly deteriorated, radial shortening showed only minor changes in patients with adverse outcomes in both cohorts. This observation points to the phenomenon that shortening in the radial direction (“bellows effect”) can relatively maintain global RV systolic function even in the face of significant longitudinal dysfunction^35^. These specific changes in the RV contraction patterns highlight the limitations of conventional, simplistic echocardiographic parameters and emphasize the added value of 3D imaging, as previous data suggest^16^.

Advanced postprocessing algorithms are now increasingly utilized in echocardiographic laboratories and elevate the quantity and quality of the reported metrics. However, in an operating theatre, these quantifications should be rather instantly available (ideally, on the echocardiographic machine) to provide “live” measurements that can guide intraoperative and immediate postoperative management. Although computational resources and developing automated or at least semiautomated methods already support these efforts well, further integration and automation are recommended to allow a wider range of health care providers to apply such advanced postprocessing.

### Study limitations

Owing to the study’s nature, our results and their interpretation are limited by several factors. All findings were observed in a single center and require external validation. This includes calculated cutoff values that are solely reflective of this specific software environment. Adverse postoperative outcomes were defined according to clinical experiences of a single center based on data of the retrospective patient cohort, instead of established endpoints from published literature. This implicates a fundamental limitation in terms of generalizability and external validation of our results. Furthermore, prospective patient inclusion resulted in a relatively small cohort. The study period was approximately six years, and changes in surgical as well as perioperative management potentially impacted the homogeneity of the data. The investigated clinical and imaging parameters included a predefined set of variables, potentially disregarding certain features of interest. Invasive hemodynamic data are not systematically available in the routine management of standard cardiac surgical cases and can therefore not be correlated with the investigated parameters. Due to these limitations, the applicability of our results to the general population is unclear.

In our study, we explored and prospectively validated the association of reduced RV longitudinal shortening with adverse perioperative outcomes in cardiac surgery patients. Our results emphasize the routine use of 3D echocardiography-derived quantification of biventricular function to enable a granular risk stratification of patients undergoing different cardiac interventions.

## Electronic supplementary material

Below is the link to the electronic supplementary material.

Supplementary Material 1

## Data Availability

Data can be obtained from the corresponding author (Dr. Marius Keller, marius.keller@med.uni-tuebingen.de) upon reasonable request and in accordance with German privacy regulations.

## Abbreviations

2D: Two-dimensional
3D: Three-dimensional
AEF: Anteroposterior ejection fraction
AIC: Akaike Information criterion
APO: Adverse postoperative outcome
CI: Confidence interval
eGFR: Estimated glomerular filtration rate
EuroSCORE: European system for cardiac operative risk evaluation
ICU: Intensive care unit
LEF: Longitudinal ejection fraction
LVEDV(i): Left ventricular end-diastolic volume (index)
LVEF: Left ventricular ejection fraction
LVESV(i): Left ventricular end-systolic volume (index)
LV GLS: Left ventricular global longitudinal strain
NYHA: New York heart association
OR: Odds ratio
REF: Radial ejection fraction
RV: Right ventricle/right ventricular
RVEDV(i): Right ventricular end-diastolic volume (index)
RVEF: Right ventricular ejection fraction
RVESV(i): Right ventricular end-systolic volume index
RV FWLS: Right ventricular free wall longitudinal strain
RV GAS: Right ventricular global area strain
RV GCS: Right ventricular global circumferential strain
RV GLS: Right ventricular global longitudinal strain
TAPSE: Tricuspid annular plane systolic excursion
TEE: Transesophageal echocardiography/echocardiograms
