# Supplementary material for: Impact of the right ventricular mechanical pattern assessed by three-dimensional echocardiography on adverse outcomes following cardiac surgery
Source: Sci Rep. 2025 Feb 15;15:5623. doi: 10.1038/s41598-025-89122-w (PMC11830065; doi:10.1038/s41598-025-89122-w)
Supplement: Supplementary file 1 — Supplementary Material 1 [file 41598_2025_89122_MOESM1_ESM.docx]

**Supplementary Figure S1: Patient selection flowchart of the retrospective cohort**

**
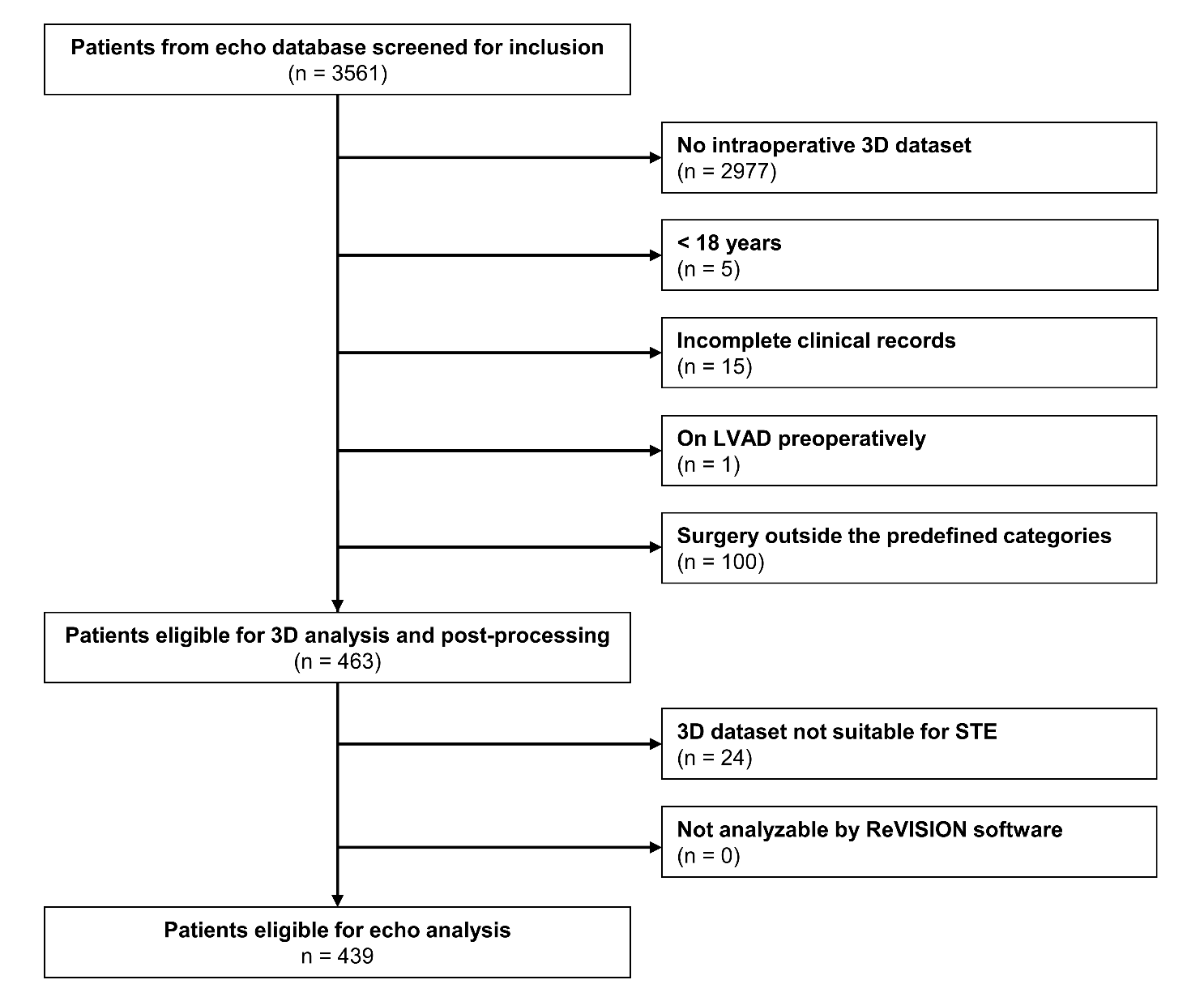
**

3D = three-dimensional, LVAD = left ventricular assist device, STE = speckle-tracking echocardiography

**Supplementary Figure S2: Patient selection flowchart of the prospective cohort**

**
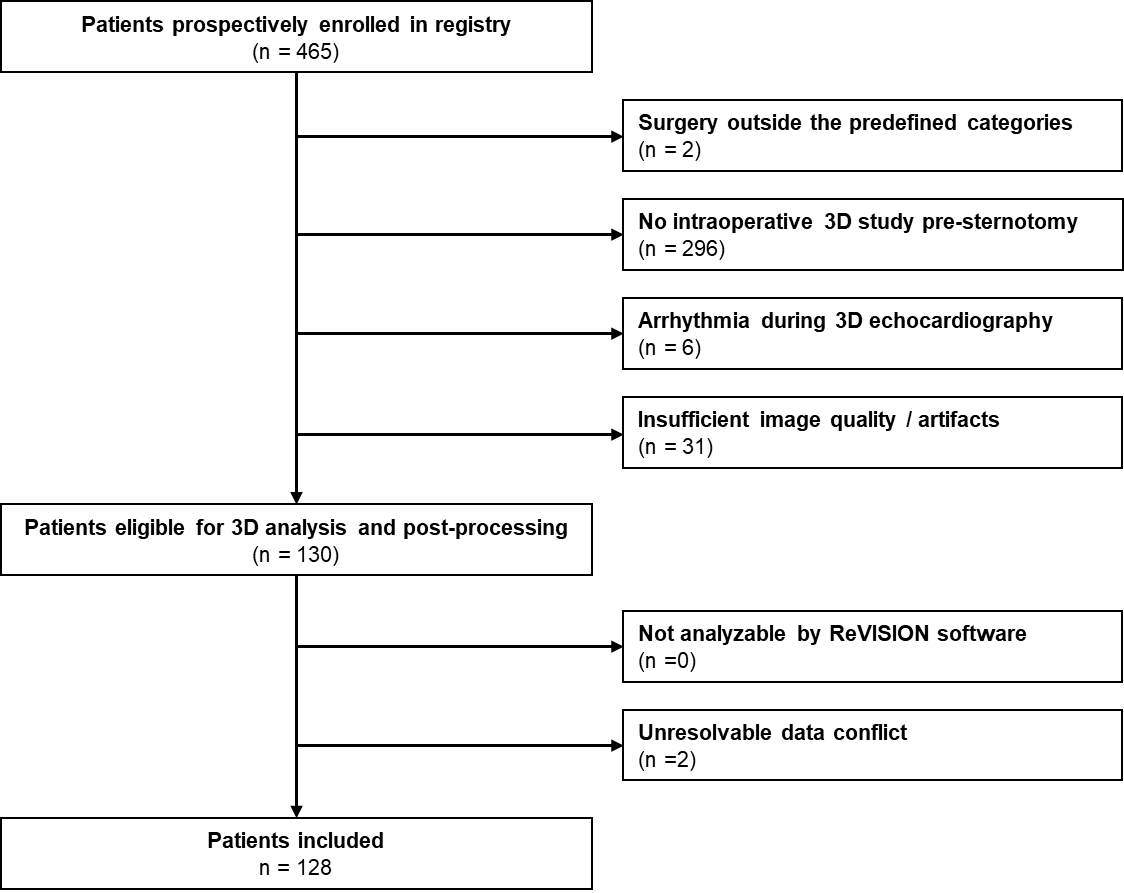
**

3D = three-dimensional

**Supplementary Table S1: Endpoint incidences and composition of adverse postoperative outcomes according to the type of surgery of the retrospective cohort**

| **Cohort** | **Composite endpoint** | **In-hospital death** | **ECLS** | **MV>48h** | **Inotropics 12h po.** | **AKI within 48h** |
| --- | --- | --- | --- | --- | --- | --- |
| **All (n=439)** | 208 (47%) | 21 (5%) | 22 (5%) | 72 (16%) | 159 (36%) | 75 (17%) |
| **On-pump coronary artery bypass grafting (n=64)** | 33 (52%) | 3 (5%) | 3 (5%) | 13 (20%) | 27 (42%) | 4 (6%) |
| **Off-pump coronary artery bypass grafting (n=92)** | 27 (29%) | 4 (4%) | 5 (5%) | 10 (11%) | 18 (20%) | 11 (12%) |
| **Left-sided valve surgery (n=122)** | 57 (47%) | 8 (7%) | 5 (4%) | 14 (11%) | 46 (38%) | 33 (27%) |
| **Thoracic aortic surgery (n=16)** | 8 (50%) | 0 (0%) | 0 (0%) | 6 (38%) | 2 (13%) | 6 (38%) |
| **Combined procedures (n=135)** | 78 (58%) | 5 (4%) | 7 (5%) | 25 (19%) | 63 (47%) | 33 (24%) |
| **Right-heart surgery (n=10)** | 5 (50%) | 2 (20%) | 2 (20%) | 4 (40%) | 3 (30%) | 2 (32%) |

AKI = acute kidney injury, ECLS = extracorporeal life support, MV = mechanical ventilation, postop. = postoperative

**Supplementary Table S2: Akaike information criterion (AIC) values of variables of clinical interest based on the univariable logistic regression analysis with p<0.1 in the retrospective cohort with regard to the composite endpoint of an unfavorable postoperative outcome**

|  | **Composite endpoint** | | |
| --- | --- | --- | --- |
| **Variable** | **OR [95% CI]** | **p-value** | **AIC** |
| **Clinical characteristics** | | | |
| **Hematocrit, %** | 0.968 [0.936-1.001] | 0.059 | 606.262 |
| **Pulmonary hypertension*** | 1.689 [0.990-2.880] | 0.054 | 607.618 |
| **Significant tricuspid regurgitation (grade ≥2)** | 2.414 [1.340-4.350] | 0.003 | **602.296** |
| **3D LV echocardiographic parameters** | | | |
| **LVEDVi, ml/m²** | 1.008 [1.002-1.014] | 0.011 | 587.504 |
| **LVESVi**, **ml/m²** | 1.012 [1.004-1.019] | 0.002 | 579.603 |
| **LVEF, %** | 0.978 [0.963-0.992] | 0.003 | **602.431** |
| **LV-GLS**, % | 1.037 [1.002-1.074] | 0.040 | 607.079 |
| **2D RV speckle-tracking echocardiography** | | | |
| **2D RV FWLS, %** | 1.032 [1.008-1.057] | 0.010 | 604.437 |
| **2D RV GLS, %** | 1.067 [1.032-1.104] | <0.001 | 596.741 |
| **ReVISION-derived 3D RV parameters** | | | |
| **RVESVi**, **ml/m²** | 1.012 [1.001-1.022] | 0.034 | 589.583 |
| **RVEF**, % | 0.962 [0.942-0.982] | <0.001 | 597.529 |
| **RV GLS**, % | 1.105 [1.059-1.153] | <0.001 | **588.595** |
| **RV GCS**, % | 1.040 [1.003-1.078] | 0.033 | 606.773 |
| **RV GAS**, % | 1.054 [1.026-1.083] | <0.001 | 595.795 |
| **LEF**, % | 0.928 [0.896-0.962] | <0.001 | 593.648 |
| **LEF/RVEF** | 0.205 [0.040-1.052] | 0.058 | 607.706 |
| **AEF**, % | 0.948 [0.918-0.980] | 0.001 | 600.821 |

2D = two-dimensional, 3D = three-dimensional, AEF = anteroposterior ejection fraction, CI = confidence interval, LEF = longitudinal ejection fraction, LV-GLS = left ventricular global longitudinal strain, LVEDV(i) = left ventricular end-diastolic volume (index), LVESV(i) = left ventricular end-systolic volume (index), LVEF = left ventricular ejection fraction, OR = odds ratio, REF = radial ejection fraction, RVEDV(I) = right ventricular end-diastolic volume (index), RVESV(I) = right ventricular end-systolic volume index, RVEF = right ventricular ejection fraction, RV FWLS = right ventricular free wall longitudinal strain, RV GAS = right ventricular global area strain, RV GCS = right ventricular global circumferential strain, RV GLS = right ventricular global longitudinal strain, , *defined as systolic pulmonary artery pressure >30mmHg

**Supplementary Table S3: Receiver-operating characteristic (ROC) analysis of 3D left and right ventricular parameters to discriminate patients of the retrospective cohort with (n=208) or without (n=231) the composite endpoint of an unfavorable postoperative outcome**

|  | **ROC analysis** | |
| --- | --- | --- |
| **Variable** | **AUC [95% CI]** | **p-value** |
| **LVEF** | 0.578 [0.524-0.632] | 0.005 |
| **LV GLS** | 0.559 [0.505-0.614] | 0.032 |
| **2D RV GLS** | 0.603 [0.550-0.655] | <0.001 |
| **3D RVEF** | 0.599 [0.546-0.652] | <0.001 |
| **3D RV GLS** | **0.629 [0.578 to 0.681]** | **<0.001** |

2D = two-dimensional, 3D = three-dimensional, AUC = area under the curve, LVEF = left ventricular ejection fraction, LV GLS = left ventricular global longitudinal strain, RVEF = right ventricular ejection fraction, RV FWLS = right ventricular free wall longitudinal strain, RV GLS = right ventricular global longitudinal strain

**Supplementary Table S4: Endpoint incidences and composition of adverse postoperative outcomes according to the type of surgery of the prospective cohort**

| **Cohort** | **Composite endpoint** | **In-hospital death** | **ECLS** | **MV<48h** | **Inotropics 12h po.** | **AKI within 48h** |
| --- | --- | --- | --- | --- | --- | --- |
| **All (n=128)** | 28 (22%) | 3 (2%) | 1 (1%) | 6 (5%) | 11 (9%) | 20 (16%) |
| **On-pump coronary artery bypass grafting (n=16)** | 5 (31%) | 0 (0%) | 1 (6%) | 0 (0%) | 3 (19%) | 2 (13%) |
| **Off-pump coronary artery bypass grafting (n=37)** | 4 (11%) | 0 (0%) | 0 (0%) | 1 (3%) | 2 (5%) | 4 (11%) |
| **Left-sided valve surgery (n=35)** | 7 (20%) | 1 (3%) | 0 (0%) | 2 (6%) | 2 (6%) | 5 (14%) |
| **Thoracic aortic surgery (n=1)** | 1 (100%) | 0 (0%) | 0 (0%) | 0 (0%) | 0 (0%) | 1 (100%) |
| **Combined procedures (n=38)** | 11 (29%) | 2 (5%) | 1 (3%) | 3 (8%) | 8 (21%) | 4 (11%) |
| **Right-heart surgery (n=1)** | 0 (0%) | 0 (0%) | 0 (0%) | 0 (0%) | 0 (0%) | 0 (0%) |

AKI = acute kidney injury, ECLS = extracorporeal life support, MV = mechanical ventilation, postop. = postoperative

**Supplementary Table S5: Akaike information criterion (AIC) values of variables of clinical interest based on the univariable logistic regression analysis with P<0.1 in the prospective cohort with regards to the composite endpoint of an unfavorable postoperative outcome**

|  | **Composite endpoint** | | |
| --- | --- | --- | --- |
| **Variable** | **OR [95% CI]** | **p-value** | **AIC** |
| **Clinical characteristics** | | | |
| **eGFR, ml/min** | 0.983 [0.966-1.001] | 0.061 | 134.776 |
| **Hematocrit, %** | 0.920 [0.841-1.007] | 0.071 | 135.221 |
| **NYHA >II** | **4.286 [1.768-10.387]** | **0.001** | **127.543** |
| **Pulmonary hypertension*** | 5.389 [1.130-25.702] | 0.035 | 134.101 |
| **Significant tricuspid regurgitation (grade ≥2)** | 2.888 [0.840-9.931] | 0.092 | 135.837 |
| **3D LV echocardiographic parameters** | | | |
| **LVESVi, ml/m²** | 1.023 [1.002-1.045] | 0.030 | 133.6932 |
| **LVEF, %** | **0.975 [0.948-1.002]** | **0.069** | **135.099** |
| **ReVISION-derived 3D RV parameters** | | | |
| **RVEDVi, ml/m²** | 1.026 [1.003-1.051] | 0.029 | 133.634 |
| **RVESVi, ml/m²** | 1.053 [1.021-1.087] | 0.001 | 126.889 |
| **RVEF, %** | **0.917 [0.871-0.964]** | **<0.001** | **125.515** |
| **RV GLS, %** | 1.176 [1.060-1.305] | 0.002 | 128.081 |
| **RV GCS, %** | 1.157 [1.050-1.276] | 0.003 | 128.674 |
| **RV GAS, %** | 1.115 [1.042-1.193] | 0.002 | 127.170 |
| **LEF, %** | 0.913 [0.843-0.990] | 0.027 | 133.360 |
| **AEF, %** | 0.896 [0.834-0.963] | 0.003 | 128.345 |
| **REF, %** | 0.928 [0.874-0.986] | 0.015 | 132.0525 |

AEF = anteroposterior ejection fraction, CI = confidence interval, LEF = longitudinal ejection fraction, LVESV(I) = left ventricular end-systolic volume (index), LVEF = left ventricular ejection fraction, OR = odds ratio, REF = radial ejection fraction, RVEDV(I) = right ventricular end-diastolic volume (index), RVESV(I) = right ventricular end-systolic volume index, RVEF = right ventricular ejection fraction, RV GAS = right ventricular global area strain, RV GCS = right ventricular global circumferential strain, RV GLS = right ventricular global longitudinal strain, *defined as systolic pulmonary artery pressure >30mmHg

**Supplementary Table S6: Multivariable logistic regression models for the composite endpoint of an unfavorable postoperative outcome in the prospective cohort**

| **Composite endpoint** | | | | | | |
| --- | --- | --- | --- | --- | --- | --- |
|  | **Model 1** | | **Model 2** | | **Model 3** | |
| **Variable** | **OR [95% CI]** | **p-value** | **OR [95% CI]** | **p-value** | **OR [95% CI]** | **p-value** |
| **Significant tricuspid regurgitation (grade ≥2)** | 2.440 [0.688-8.646] | 0.167 | 2.441 [0.599-8.388] | 0.231 |  |  |
| **LVEF** | 0.978 [0.950-1.005] | 0.113 |  |  | 0.988 [0.959-1.018] | 0.421 |
| **(3D) RV GLS** |  |  | 1.165 [1.050-1.293] | 0.004 | 1.158 [1.039-1.292] | 0.008 |

3D= three-dimensional, CI = confidence interval, LVEF = left ventricular ejection fraction, OR = odds ratio, RV GLS = right ventricular global longitudinal strain

**Supplementary Table S7: Multivariable logistic regression models for the composite endpoint of an unfavorable postoperative outcome in the prospective cohort**

|  | **Composite endpoint** | | | | | | | |
| --- | --- | --- | --- | --- | --- | --- | --- | --- |
|  | **Model 1** | | **Model 2** | | **Model 3** | | **Model 4** | |
| **Variable** | **OR [95% CI]** | **p-value** | **OR [95% CI]** | **p-value** | **OR [95% CI]** | **p-value** | **OR [95% CI]** | **p-value** |
| **NYHA >II** | 3.248 [1.277-8.211] | 0.013 | 3.997 [1.632-9.787] | 0.002 | 3.288 [1.301-8.309] | 0.012 |  |  |
| **LVEF** | 0.990 [0.959-1.021] | 0.519 | 0.980 [0.951-1.009] | 0.169 |  |  | 0.988 [0.959-1.018] | 0.421 |
| **(3D) RV GLS** | 1.124 [1.004-1.259] | 0.042 |  |  | 1.138 [1.022-1.267] | 0.019 | 1.158 [1.039-1.292] | 0.008 |

3D= three-dimensional CI = confidence interval, LVEF = left ventricular ejection fraction, OR = odds ratio, RV GLS = right ventricular global longitudinal strain
